# Editorial: Myopia: Public health challenges and interventions

**DOI:** 10.3389/fpubh.2022.1008858

**Published:** 2022-09-14

**Authors:** Rafael Iribarren, Andrzej Grzybowski, Carla Lanca

**Affiliations:** ^1^Drs. Iribarren Eye Consultants, Buenos Aires, Argentina; ^2^Department of Ophthalmology, University of Warmia and Mazury, Olsztyn, Poland; ^3^Institute for Research in Ophthalmology, Foundation for Ophthalmology Development, Poznan, Poland; ^4^Escola Superior de Tecnologia da Saúde de Lisboa (ESTeSL), Instituto Politécnico de Lisboa, Lisboa, Portugal; ^5^Comprehensive Health Research Center (CHRC), Escola Nacional de Saúde Pública, Universidade Nova de Lisboa, Lisboa, Portugal

**Keywords:** myopia, public health, confinement, COVID-19, lockdown, screen time

Most school myopia results from an excessive axial length of the eye that develops in childhood. In the past three decades there were significant increases in the prevalence of childhood myopia. By 2050, half of the world population is expected to have myopia, a 2-fold increase compared to year 2000 (1). In the last years, the achievements made by scientists have been exceptional, leading to major advancements in the treatment of myopia progression. This Research Topic comprises 14 studies including original research articles and reviews covering several aspects related with myopia.

Myopia has become one of the fastest-growing eye health challenges of the twenty-first century, with a disproportionate burden on urban Asia regions (2). Shi et al. conducted a study on temporal and spatial characterization of myopia in China. The authors showed that there was an increase in the prevalence of myopia in children aged 7–18 years old from 1995 to 2014. The study results also showed a shift of myopia to the southeast, identifying the existence of high-risk areas. Those results are important for targeted myopia prevention.

Myopia is a leading cause of visual impairment and blindness across many countries (3). Many myopic adolescents with high myopia today may be legally blind due to myopic maculopathy in 30 years' time. Considering the significant burden of the disease and its complications, tackling myopia becomes imperative. Thus, it is important to understand risks factors for myopia, to develop appropriate prevention plans and treatment strategies. The evidence for the association between sleep and myopia or gender and myopia has been mixed. Li, Tan et al. showed that sleep quality, duration, timing, and the consistency of specific sleep factors were not associated with myopia among school-aged children. Xu et al. reported that puberty status among adolescents may be an independent risk factor for myopia in girls but not boys, suggesting that earlier puberty in girls explained a significant proportion of the sex disparity in myopia prevalence. However, detailing the public health implications of both study findings requires further longitudinal studies with more accurate measures of sleep and puberty status.

The pandemic lockdowns established by the authorities for curving COVID-19 pandemic led to detrimental effects on myopia development due to a significant decrease in outdoor time and increase in near work activities (4, 5). Limwattanayingyong et al. reviewed the evidence supporting the association between environmental and social factors and myopia resulting from the COVID-19 Pandemic. The authors found sufficient evidence to support the association between an increase in near work from home confinement or a reduction of outdoor activities and worsening of myopia during the COVID-19 lockdown. The findings from this review may help to better understand myopia development and progression, and lead to recommendations to prevent myopia and its progression. Efforts to reduce the prevalence, progression, and severity of myopia could have a profound impact on public health. Keel et al. propose a digital message program named “WHO-ITU MyopiaEd Programme” targeting education on myopia and its prevention. The program aims to strengthen countries' efforts to develop sustainable, cost-effective, and acceptable activities to support education on myopia and its prevention. Those programs may need to be implemented taking in consideration the diversity of eye care behaviors among adolescents. According to Li, Wang et al. there are differential profiles related with basic demographic characteristics and visual acuity development. Personalized group intervention for students in different latent classes behaviors may enhance the intervention results.

Myopia is a chronic condition where the evidence is changing at an accelerated pace and 1,000's of research studies about myopia have been published within the last 100 years. Shan et al. conducted a bibliometrics analysis to help researchers to comprehend the global trends of myopia research from 1900 to 2020. Research Topics were clustered into six groups, with “prevalence and risk factors of myopia” and “surgical control of myopia” being the largest groups with higher number of publications. With the increasing prevalence of myopia, interventions to control myopia progression are a potential research hotspot and pressing public health issue. Shinojima et al. conducted a mini review on the current evidence-based treatments for myopia progression, such as atropine eye drops, optical treatment with defocus and orthokeratology. New research with optical treatments also showed good efficacy in the control of myopia progression. However, there are other factors that need to be considered, such as the uptake by eye care practitioners that can be improved if more education is given. Yang et al. conducted a study on eye care practitioners and their influence in prescribing myopia control. The authors found that the cost of myopia control is of concern to eye care practitioners. Further research is also required to establish the minimum age, amount of myopia, and progression to start prescribing myopia control interventions.

Previous epidemiological research on myopia has been mainly focused on school-age children. However, it is essential to identify children at high risk of developing myopia to prevent myopia in an early stage, especially during the preschool period. The findings of a cross-sectional study by Matsumura et al. outline the importance of obtaining an accurate family history of myopia to identify at-risk children before they develop myopia and to raise awareness on lifestyle-based myopia prevention. You et al. analyzed longitudinal changes in refractive error among preschool children and found a myopic shift of 0.20 D on average per year. The most important change in spherical equivalent occurred in 3-years-olds prompting the need for more prospective studies to better explain the factors related to refractive status changes and to prevent myopia in preschool children.

Tao et al. suggest that during the growth of school-age children, a significant correlation exists between axial length and height, and between axial length growth and height growth, especially in children with newly developed myopia. This indicates that during the period of rapid height growth, the elongation of axial length also needs to be considered. On the other hand, Lee and Mackey reviewed the findings from the Raine study in young adults with myopia. The results support that myopia can progress in the third decade of life, with some individuals progressing at alarming rates. Thus, it is also critical that longitudinal birth cohort studies in other populations increase their focus on research in young adults. Lan et al. showed that about half of the interviewed adults patients believed laser refractive surgery could cure myopia and its complications. The results of this study show that patients with myopia need to receive more education on laser refractive surgery and rhegmatogenous retinal detachment to increase early detection and potentially prevent the disease complications.

To prevent myopia and its complications it is essential to unravel the causes that have produced the myopia epidemic in East and Southeast Asian urban environments. More research is necessary on the lack of outdoor exposure since an early age in childhood, and early high academic load of more than 10 h of schooling a day 6 days a week with short annual vacations, at which Asian children in many urban cities are exposed. The manuscripts published in this Research Topic show that the myopia epidemic has occurred along with urbanization and that myopia develops early since kindergarten years, continuing to progress in young adulthood. Recent changes in the tutorial classes education system by the government in China have been accepted by the society but myopia education programs are welcome to prevent myopia. One of the main concerns about myopia control is the cost effectiveness of the new available treatments. Thus, much has still to be learned and we hope the Research Topic of studies presented in this Research Topic of Frontiers in Public Health inspires, informs, and provides directions and guidance to governments and researchers in the field of myopia.

## Author contributions

CL prepared the original draft. RI and AG critically reviewed and edited the manuscript. All authors have reviewed and approved the final manuscript.

## Conflict of interest

All authors declare that the research was conducted in the absence of any commercial or financial relationships that could be construed as a potential conflict of interest.

## Publisher's note

All claims expressed in this article are solely those of the authors and do not necessarily represent those of their affiliated organizations, or those of the publisher, the editors and the reviewers. Any product that may be evaluated in this article, or claim that may be made by its manufacturer, is not guaranteed or endorsed by the publisher.
